# Phenylazopyridine as Switch in Photochemical Reactions. A Detailed Computational Description of the Mechanism of Its Photoisomerization

**DOI:** 10.3390/ma10121342

**Published:** 2017-11-23

**Authors:** Josep Casellas, Gerard Alcover-Fortuny, Coen de Graaf, Mar Reguero

**Affiliations:** 1 Departament de Química Física i Inorgànica, Universitat Rovira i Virgili, Carrer Marcel·lí Domingo 1, 43007 Tarragona, Spain; josep.casellas@urv.cat (J.C.); gerard.alcover@urv.cat (G.A.-F.); coen.degraaf@urv.cat (C.d.G.); 2 Institució Catalana de Recerca i Estudis Avançats (ICREA), Passeig Lluís Companys 23, 08010 Barcelona, Spain

**Keywords:** phenylazopyridine, photochromism, photoisomerization, CASSCF/CASPT2, reaction mechanism

## Abstract

Azo compounds are organic photochromic systems that have the possibility of switching between *cis* and *trans* isomers under irradiation. The different photochemical properties of these isomers make azo compounds into good light-triggered switches, and their significantly different geometries make them very interesting as components in molecular engines or mechanical switches. For instance, azo ligands are used in coordination complexes to trigger photoresponsive properties. The light-induced *trans*-to-*cis* isomerization of phenylazopyridine (PAPy) plays a fundamental role in the room-temperature switchable spin crossover of Ni-porphyrin derivatives. In this work, we present a computational study developed at the SA-CASSCF/CASPT2 level (State Averaged Complete Active Space Self Consistent Field/CAS second order Perturbation Theory) to elucidate the mechanism, up to now unknown, of the *cis–trans* photoisomerization of 3-PAPy. We have analyzed the possible reaction pathways along its lowest excited states, generated by excitation of one or two electrons from the lone pairs of the N atoms of the azo group (n_azo_π*^2^ and n_azo_^2^π*^2^ states), from a π delocalized molecular orbital (ππ* state), or from the lone pair of the N atom of the pyridine moiety (n_py_π* state). Our results show that the mechanism proceeds mainly along the rotation coordinate in both the n_azo_π* and ππ* excited states, although the n_azo_^2^π*^2^ state can also be populated temporarily, while the n_py_π* does not intervene in the reaction. For rotationally constrained systems, accessible paths to reach the *cis* minimum along planar geometries have also been located, again on the n_azo_π* and ππ* potential energy surfaces, while the n_azo_^2^π*^2^ and n_py_π* states are not involved in the reaction. The relative energies of the different paths differ from those found for azobenzene in a previous work, so our results predict some differences between the reactivities of both compounds.

## 1. Introduction

Azo compounds constitute a well-known organic family with photochromic properties due to the easy interconversion between their *trans* and *cis* isomers, which have different absorption spectra. The parent system—azobenzene— and its derivatives have a wide range of applications due to the possibility to tune their properties through substitutions to control the rates of the forward and back reactions, their fatigue resistance, and their readiness to be incorporated in different kinds of material [1,2]. 

**Scheme 1:**
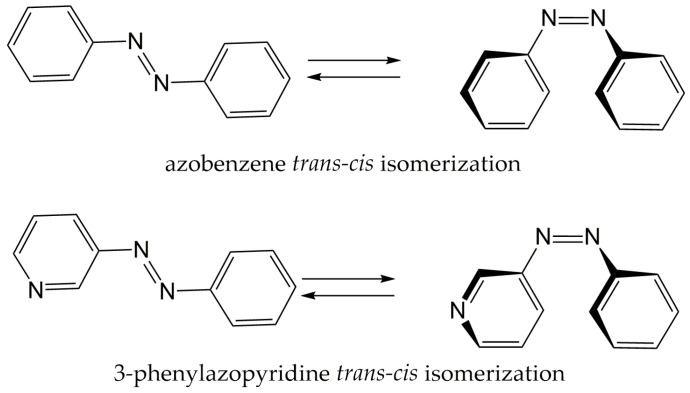
Azobenzene and 3-phenylazopyridine (3-PAPy) *trans*-*cis* isomerization reactions.

Given its role as a molecular switch, azo compounds have also been used in coordination complexes as a trigger of photoresponsive properties. In particular, the light-induced *trans*-to-*cis* isomerization of phenylazopyridine (PAPy) (Scheme 1) plays a fundamental role in the room-temperature switchable spin crossover of Ni-porphyrin derivatives. One of these complexes, where the photoreactive ligand is the 3-PAPy, has already been investigated by our group [3]. In this photochemical process, the lone pair on the nitrogen of the pyridine can be coordinated or not to a nickel ion, depending on the orientation of the PAPy arm imposed by the *trans* or *cis* isomer, changing the coordination number of the metal centre and consequently its spin multiplicity, leading to a reversible spin crossover. 

Although PAPys have been extensively studied for decades in the synthesis and characterization of metallic complexes, the study of photoisomerizable ligands and the light-induced processes that they give rise to has only recently started. For this reason, we have devoted this work to elucidating the mechanism of photoisomerization of 3-PAPy, to obtain information that can be used to tune the photochemical properties of this family of compounds. The study will be conducted using mainly ab initio multiconfigurational computational methods: in particular, the SA-CASSCF/MS-CASPT2 methodology (State Averaged Complete Active Space Self Consistent Field/CAS second order Perturbation Theory).

In most works, the photochemical properties of PAPys are compared with those of their extensively studied parent system, azobenzene, so it seems convenient to give first a brief abstract of the knowledge heretofore gathered about this latter compound. 

**Figure 1:**
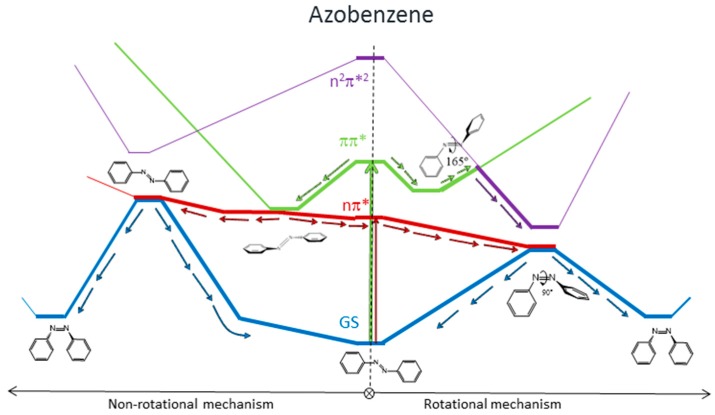
Schematic representation of the lowest energy potential energy surfaces (PES) of azobenzene and the proposed reaction mechanisms for systems with free and constrained rotation proposed in reference [8] for azobenzene.

The absorption spectrum of *trans*-azobenzene shows two bands in the UV-visible region. The first one is a weak band located at 430 nm, which populates the S_1_ state of nπ* character. The second band, much stronger, is found at 320 nm and populates the S_2_ state of ππ* character. When these states are populated separately, higher isomerization quantum yields are obtained when the system is excited to the nπ* state [4,5], which suggests that the mechanisms to reach the *cis* isomer are different depending upon the state along which the reaction takes place. The mechanisms traditionally proposed are pure rotation and inversion (although combinations of both mechanisms have also been suggested later on). In the first mechanism, one of the phenyl groups moves out of the plane of the molecule, changing the C–N–N–C dihedral angle from 180° to 0°. On the other hand, inversion implies the in-plane movement of a phenyl ring, modifying one of the N–N–C angles from 120° to 240°. This mechanism would explain why photoisomerization can also take place in systems with restricted rotation, and why the quantum yield of the reaction is always the same in those cases, independent of the excited state populated by the initial irradiation [6,7]. Recently, our group developed a computational study at the SA-CASSCF/CASPT2 level to explain the mechanism of this reaction [8], the main results of which are represented in Figure 1. We found that for systems with free rotation, photoisomerization from the nπ* state follows a rotational mechanism. The system relaxes along the nπ* surface to the rotated minimum and decays to the ground state through a conical intersection located nearby. Once on the ground-state surface, the system can evolve back to the more stable *trans* isomer, or forward to the *cis* isomer following the inertia of the movement of the nuclei. If the excitation takes the systems to the ππ* state, there are two possible relaxation coordinates on this surface. The main one leads to a pedal-like minimum. At this geometry, ππ* and nπ* states are almost degenerate, so the deactivation to the nπ* state will be fast and efficient. The subsequent evolution of the system will continue along the latter surface, following the path explained before. The second possible relaxation coordinate on the ππ* surface leads to a local slightly rotated minimum, near which a crossing with the n^2^π*^2^ is located. Decay to this surface would lead to the minimum energy geometry of this state, a rotated structure, where deactivation to the nπ* state and eventually to the ground state is easy.

For systems with restricted rotation, the photoisomerization along the nπ* surface follows a concerted inversion pathway. Along this path, a planar conical intersection with the ground state is found. Like in the previous case, deactivation to the ground state can lead back to the reactant or to the photoproduct. This path is less accessible than the rotational one, given that the planar nπ*/S_0_ conical intersection is located at higher energies than the rotated nπ*/S_0_ one. If the rotationally restricted system is excited to the ππ* state, only relaxation to the pedal-like minimum will be possible. From here, after internal conversion to the nπ* state, the system will decay to the ground state through the planar nπ*/S_0_ conical intersection (CI). This funnel will be accessed more easily after an excitation to the ππ* state than when the system is excited to the nπ* state since the absorbed initial energy necessary to reach it and the residual energy are larger than the amount necessary to reach the nπ* state. 

In spite of the similarities between azobenzene and phenylazopyridine, the presence of an extra nitrogen atom in the latter compound gives rise to new excited states (involving excitations from the lone pair (n) electrons of this heteroatom, generating n_py_π* excited states) that can be of low energy and intervene in the reaction, modifying its mechanism. On top of this, given that the energy differences between the states and structures that play a role in the photoisomerization mechanism of azobenzene are not large, small quantitative energy changes can invert relative stabilities and produce noticeable qualitative changes in the reaction mechanisms for these systems, which is a reason why specific studies on PAPys are considered necessary. 

The first mechanistic study of the photoisomerization of these compounds did not appear until 2009 when Wang [9] characterized in detail the potential energy surfaces of the ground and first excited states for 2-PAPy and 4-PAPy. Their results indicated that photochemistry of 4-PAPy was very similar to that of azobenzene, but subtle differences were predicted for 2-PAPy in the path of rotation on the nπ* state where a faster decay was observed. On the other hand, no mechanistic studies have been published for 3-PAPy, to our knowledge, up to now; for this reason, we have developed this study. 

The main questions that we will address are those alluded to in the previous paragraph: Does the low-lying n_py_π* excited state play a role in the photochemistry of 3-PAPy? Are there significant differences between the photoisomerization mechanisms of 3-PAPy relative to that of azobenzene? Our results will show that, although the n_py_π* excited state does not play any role in the photoisomerization mechanism of 3-PAPy, the small quantitative energy changes between this system and azobenzene can induce noticeable changes in the photochemistry of both systems.

## 2. Computational Strategy

### 2.1. Potential Energy Surfaces and Elucidation of the Reaction Mechanism

The study of the mechanistic profiles of phenylazopyridine was conducted following the SA-CASSCF/MS-CASPT2 protocol (where SA-CASSCF stands for State Averaged Complete Active Space Self Consistent Field; MS for Multistate and CASPT2 for CAS corrected at second order of Perturbation Theory). The SA-CASSCF method was used for geometry optimizations in the states that did not need the effect of the dynamical electron correlation in order to be correctly described. This is the case for the ground state, the double-excited n^2^π*^2^ state, and the n_py_π* state. On the stationary points located on these surfaces, the energetics were refined with the MS-CASPT2 method. In the case of the nπ* and ππ* states, MS-CASPT2 was used for both geometry optimizations and relative energy calculations, given that our experience in the previous study on azobenzene [8] showed that these states are not well described at the SA-CASSCF level.

To include the whole π system of PAPy in the active space, it would be necessary to take into account 18 electrons in 16 orbitals. Unfortunately, an active space of this size is unaffordable in standard calculations, so the active space was reduced by excluding those orbitals for which occupation did not change noticeably from two or from zero in the regions of the potential energy surfaces (PES) explored of the states considered. The result was an active space of 12 electrons in 9 orbitals, composed of two π and two π* orbitals of the benzenes, the π and π* of the azo group, and the orbitals of the lone pairs of the nitrogen atoms (see Figure S1 of Supplementary Materials). Test calculations showed that bigger active spaces do not give noteworthy differences. A Pople d-polarized split-valence basis set 6-31G (d) was used for all calculations; larger basis sets only cause small differences in the vertical excitation energies (see Figure S2 of the Supplementary Materials).

The reference wavefunctions and the molecular orbitals were obtained by state-average SA-CASSCF calculations. In order to explore all the possibilities of the mechanism, symmetry constraints were not used although *trans*-PAPy presented a C_2h_ geometry. The topography of the PES between critical points of interest was explored using the linear interpolation of internal coordinates strategy (LIIC). Minimum energy paths (MEP) were also computed. In all the energetics, an imaginary shift of 0.1 was added for the zero-order Hamiltonian in the MS-CASPT2 calculations in order to preclude the inclusion of intruder states [10]. The CAS state interaction (CASSI) protocol was used to compute the transition oscillator strengths in order to compare transition probabilities among the different studied states [11]. Frequency calculations at the CASSCF level were also run to determine the nature of the stationary points at this level. When necessary, grids of CASPT2 calculations around CASSCF-optimized geometries were run to confirm their minimum character at the CASPT2 level. Frequency calculations and the MS-CASPT2 optimizations were done numerically due to the lack of implementation of analytical gradients with this protocol. To make these calculations affordable, Cholesky decomposition of the two-electron integral matrix was used [12]. All calculations were performed using MOLCAS 7.6 (Lund, Sweden) package [13].

Conical intersections and transition states (TS) were optimized using the algorithm implemented in Gaussian 09 (Gaussian, Inc., Wallingford, CT, USA) [14]. For the former, state-averaged orbitals were used and the orbital rotation derivative correction to the gradient (which is usually small) was not computed. The full diagonalization method was suppressed in order to afford these calculations, which provide the lowest energy point of the crossing. 

### 2.2. Simulation of the Absorption Spectrum

There are several computational strategies to calculate accurate excitation energies, but this is not sufficient to obtain a full simulation of the UV-vis absorption spectrum. The basic problem is that, in the “idealized” and simplified determination of the PES, the deformations of the stationary geometries due to the thermal energy are not contemplated. Consequently, for systems with structural symmetry, the transition dipole moments (governed by selection rules) between some states are strictly zero by symmetry. However, in experimental conditions, the geometry deformations due to the thermal energy break the symmetry of the system, making those ‘forbidden’ transitions possible. Consequently, the experimental spectra show more bands than those predicted computationally. This has already been observed before in other systems (as an example, see the case of nitrobenzene in reference [15]) and is also the case of the system studied here, because the minimum energy geometry is planar for the most stable *trans* isomer and, for example, nπ* transitions have strictly zero transition dipole strength. For this reason, in order to reproduce the absorption spectrum, we have used molecular dynamics as a consistent geometry generator to statistically represent 3-PAPy in a real spectroscopic environment at room temperature (298 K).

For this purpose, we run classical molecular dynamics (MD) with periodic boundary conditions using the AMBER 99 force field [16], as implemented in GROMACS 4.5 [17]. A simulation box of size 28 × 31 × 38 Å was used to avoid interactions between the images of the 3-PAPy molecule, which is surrounded by 513 methanol molecules, corresponding to a concentration of approximately 0.05 M [18]. The parameters of the force field that determine the topology of the methanol molecule are shown in the Supplementary Materials.

Equilibration runs were performed in the NVT (isothermal-isochoric) ensemble (100 ps) and subsequently in the NPT (isothermal-isobaric) ensemble (100 ps), controlling the temperature and pressure with the Berendsen coupling algorithm where the reference temperature and pressure were established at 298 K and 1 bar, respectively. Upon completion of the two equilibration phases, a final 100 ps MD simulation was run at constant temperature. 

Using a code designed in our group [19], the vertical energies and transition dipole strengths of the nine lowest excited states were computed at 400 snapshots along the MD trajectory using the SA-CASSCF/CASPT2 protocol, introducing the effect of the solvent (methanol) with the polarizable continuum model (PCM). 

## 3. Results and Discussion

The study of any photochemical process can be separated into distinct stages: initial excitation (to compute the probability to populate different excited states); subsequent possible relaxations along the PES of the excited states populated; and, finally, secondary processes that can occur afterwards. This sequential structure is going to be followed in the exposition of these results. First of all, we analyze the ground-state stable structures. In the second subsection, we collect the results for the absorption spectrum. In the third part, we analyze the initial relaxation on each excited-state surface through the accessible pathways. In the last subsection, we present the possible subsequent events, which in some cases correspond to competitive processes. All this information will allow us to propose a comprehensive mechanism for the photoisomerization of 3-PAPy, which is also presented in this last subsection.

Throughout this section, we will compare the results obtained for phenylazopyridine with those of azobenzene, pointing out the mechanistic consequences that the differences between both systems can imply. All the energies given in this section correspond to MS-CASPT2 results unless explicitly indicated otherwise.

### 3.1. Franck–Condon (FC) Region

**Figure 2:**
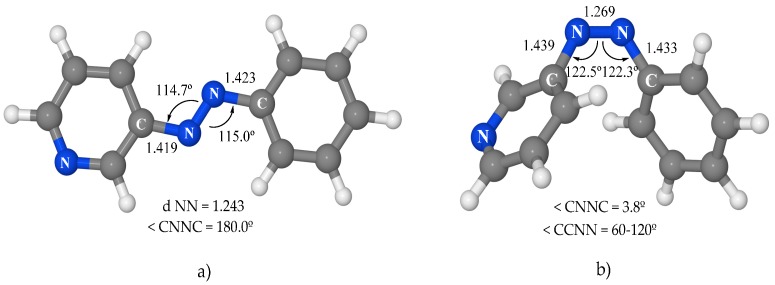
(**a**) Ground-state global minimum; (**b**) *cis* isomer minimum. Both optimized at the SA-CASSCF level. dNN: N–N distance; < CNNC: dihedral angle C–N–N–C. All distances are in Å.

The optimization of the structural parameters for the ground-state minima is the first step of this study. The asymmetry of the pyridine ring gives rise to two possible conformers for each isomer. Only the more stable one will be considered in this work. The minima for the *trans* and *cis* isomers obtained (Figure 2) are very similar to those of azobenzene, except for the asymmetry between the phenyl and pyridine moieties. In *trans*-3-PAPy, the NC bonds and NNC angles are slightly smaller in the pyridine moiety than in the phenyl one (1.419 Å and 114.7° in the first versus 1.423 Å and 115.0° in the second). In *cis*-3-PAPY, the structural parameters are remarkably similar to those of azobenzene. From the energetic point of view, the *cis* isomer is 11.3 kcal·mol^−1^ above the global *trans* minimum; this energy difference is 3.0 kcal·mol^−1^ lower than that in azobenzene. 

The analysis of the wavefunctions of the singlet excited states at the *trans* geometry (vertical excitations) shows the nature of the low-energy excited states, that we represent as nπ*, n_py_π*, ππ*, and n^2^π*^2^. The n, π, and π* orbitals involved in the excitations correspond mainly to combinations of orbitals located on the N atoms of the azo group, although the π orbital also shows some participation of the π systems of the rings. On the other hand, the n_py_ orbital shows almost exclusive contribution from the n orbital of the pyridine N atom (see Figure S1 of Supplementary Materials). The main difference with azobenzene is the existence of a n_py_π* state. At the SA-CASSCF level, this state appears as the eighth root, but the CASPT2 treatment shows that the dynamic electron correlation is a determinant for the description of this state, which is, in fact, the second excited state of the Franck–Condon geometry. Given its position in between the lowest energy states of nπ* and ππ* character (Table 1), its possible involvement in the mechanism of photoisomerization has to be explored.

As can be seen in Table 1, the experimental energy for the n_py_π* state is quite different to the calculated value. This disagreement could be due to the flexibility of the C–N–N–C dihedral angle in solution, because this coordinate is extremely unfavorable for the n_py_π* state, so small changes in this variable will induce large changes in energy. Another discrepancy between computational and experimental results is found in the predicted intensities of the absorption bands, given that, despite the low oscillator strength calculated for excitations to the nπ*and n_py_π*states, these bands are present with a noticeable intensity in the spectra (see Table 1). The reason for this disagreement could also lie in the flexibility of the compound, which easily breaks the planarity, decreasing the symmetry of the system and allowing some transitions that are forbidden for the planar species—the only species considered in Table 1 for *trans*-3-PAPy. To check this hypothesis, our group considered it interesting to try to reproduce with greater accuracy the experimental absorption spectra of this compound. 

### 3.2. Simulation of the Absorption Spectrum 

In this study, we compare the spectrum computed for 3-PAPy with the scarce experimental spectroscopic data available for this system. As commented upon in the Computational Details section, to generate this spectrum we have computed (at the MS-CASPT2 level) the vertical energies and oscillator strengths of a number of geometries that were selected from a molecular dynamics calculation, in order to gather a statistical representation of the molecular behavior of a sample of *trans*-3-PAPy 0.05 M in methanol at room temperature. 

In order to be sure that we are not cherry-picking the geometries that seem to give the expected results, the average value and the variation were checked for the C–N–N–C dihedral angle, C–N–N angle, and N–N distance. Their distributions on the set of conformations that form the sample (shown in Figure S3 of Supplementary Materials) give average values similar to those of the planar minimum (although they are not exactly centred around those values), which indicates that the geometries selected properly represent the variability of the structural modifications in a real system. 

**Figure 3:**
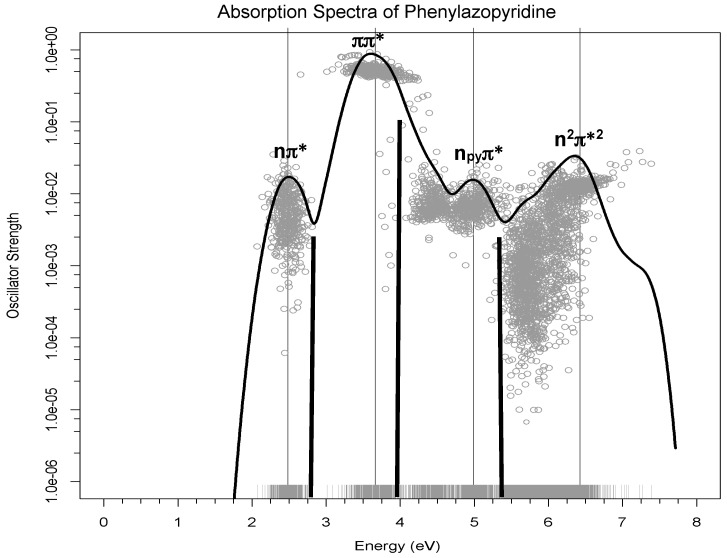
Theoretical simulation of the absorption spectrum of 3-PAPy in methanol. Bold lines correspond to the maxima of the bands (energies and intensities) of the experimental spectrum [18].

At each geometry, the energies of the 10 most stable states were calculated, taking into account the environment of the solvent by the PCM method together with the transition dipole moment from the ground state. Each one of these calculations then provides the energy gap and oscillator strengths for 9 transitions for each geometry. The oscillator strengths (proportional to the experimental intensity of the transition) versus the transition energies for these 3600 transitions (400 geometries × 9 transitions) are represented in Figure 3 as grey dots. In order to sum up all absorptions, an R code was used to build the Gaussian curves using the accumulated intensities of each transition. The resulting black curve corresponds to a theoretical estimate of the experimental absorption spectrum. The low energy bands are labelled according to the excited states involved in the transition. The black bold lines represent the energy and intensity of the maxima of the experimental spectrum [18]. It should be pointed out that there is no spectroscopic information for high energy bands, so the fourth band predicted theoretically has no experimental counterpart. 

This figure shows that the three-band shape in the range of energies of the experimental spectrum [18] is well reproduced, although there is a regular shift of 0.3 eV in the position of the maxima of the bands, as can be observed from the data collected in Table 2. To analyze if this difference was due to the modelization of the solvent as a continuum, test calculations were performed including explicit methanol molecules in the model described at quantum level. The small change in the maxima of the bands showed that this was not the reason for the gap. 

Comparison with the absorption energies of the single-point calculation at the Franck–Condon geometry (also collected in Table 2) shows that the single-point energies are larger than the computed band maxima for the nπ* and ππ* transitions, while the opposite is true for the n_py_π* transition. The reason for this difference can be explained by the trend of the variation of energy with the main deformations from the equilibrium geometry of the sample. In fact, we have already seen that, in general, the internal coordinate that most affects the energy is the dihedral angle C–N–N–C. In the ground-state minimum, this angle is 180° (planar structure), but the distribution of values of this parameter in the collective that reproduces the experimental sample (Figure S3 of Supplementary Materials) shows that, in a large percentage of geometries, the deviation is more than 5°, with the mode being around 175°. For ππ* and nπ*, this variation in the dihedral angle leads to a decrease in the energy, while for n_py_π* the effect is the opposite and more pronounced. Consequently, the average excitation energy of the collective to the first states will be lower than for the planar ground-state minimum, while it will be higher for the n_py_π* state, and will show a larger difference. The disagreement found between the computational and experimental data for the n_py_π* transition can then be explained by the simplification made when considering a single geometry to calculate the excitation and compare with the maximum of the band, together with the strong dependence of the energy on a geometrical parameter (the dihedral angle in this case).

### 3.3. Minimum Energy Points on Excited-State Surfaces

**Figure 4:**
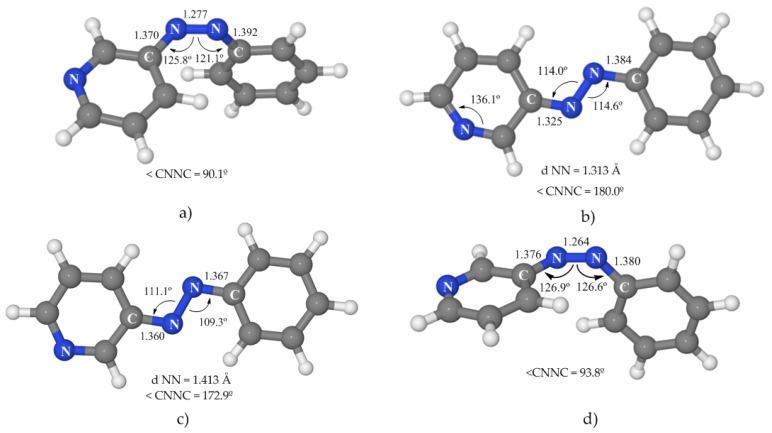
Minima of the excited states: (**a**) ^1^(nπ*) state minimum optimized at the MS-CASPT2 level; (**b**) ^1^(n_py_π*) state minimum optimized at the SA-CASSCF level; (**c**) ^1^(ππ*) state minimum optimized at the MS-CASPT2 level; (**d**) ^1^(n^2^π*^2^) state minimum optimized at the SA-CASSCF level.

The PES of the states ^1^(nπ*), ^1^(n_py_π*), ^1^(ππ*) and ^1^(n^2^π*^2^) have been examined. Each state has been optimized following the protocol previously explained. Although major structural differences between azobenzene and 3-PAPy are not expected, the involvement of the n_py_π* state and changes in relative energies can modify the balance between states that eventually lead to different photochemical processes. Obtained geometries and computed energies are shown in Table 3 and Figure 4, and specific comments on each state are to be found below.

#### 3.3.1. ^1^(nπ*) State

Like in the case of azobenzene, SA-CASSCF does not properly describe this state, and the minimum energy geometry obtained at this level (a planar structure, see Figure S4 of Supplementary Materials) does not correspond to the absolute minimum. When the geometry was optimized at the MS-CASPT2 level, a deeper minimum was detected that shows a rotated geometry with a C–N–N–C dihedral angle of 90°, asymmetric CNN angles, and a clearly shortened N–N distance (Figure 4a). This CASPT2 minimum is placed 48.4 kcal·mol^−1^ above the ground-state minimum, around 15 kcal·mol^−1^ lower than the vertical excitation energy to this state (see Table 3).

As can be seen in Table 3, the values for the n_py_π* state are not shown for rotated geometries, because rotation represents a highly unfavorable coordinate for this state, destabilising it so much that this state does not correspond to any of the roots calculated.

#### 3.3.2. ^1^(n_py_π*) State

The minimum obtained for this state was optimized at the SA-CASSCF level and confirmed at the CASPT2 level by the calculation of a grid modifying the most important geometrical parameters of this PES around the CASSCF-optimized geometry. This minimum was found to be 75.7 kcal·mol^−1^ above the ground-state minimum (see Table 3). Although it is located on the S_1_ PES, the high energy of this species indicates that it is quite probable that this state will not be involved in the photoisomerization mechanism of 3-PAPy. The stabilization of this state relative to the ^1^(nπ*) and ^1^(ππ*) states is mainly due to the strong deformation that occurs in the pyridine ring, where the C–N_py_–C angle enlarges from 117.7° in the FC geometry to 136.1° in the optimized geometry (Figure 4b). This modification can be understood in the context of the valence-bond theory where the N is expected to have sp^2^ hybridization in the ground state while the hybridization of this atom is closer to sp in the n_py_π* state due to delocalization of one electron of the lone pair and, consequently, the C–N_py_–C angle opens. Even though these changes are noticeable, the structure continues being planar, with an elongated N–N distance and a much shorter N_azo_–C_py_ bond, while the N_azo_–C_bz_ distance hardly changes. 

We must point out that before reaching the minimum, this state becomes the first excited state and, therefore, there must be a crossing with the nπ* state during the in-plane relaxation process.

#### 3.3.3. ^1^(ππ*) State

3-PAPy shows two ππ* states of low energy in rotated geometries, corresponding to the excitation of an electron from a molecular orbital located on the phenyl or on the pyridine rings. In each case, the lower excited state has been selected for this study.

Taking into account the results on azobenzene, we expected that the description of this state at the SA-CASSCF level was not reliable, so the geometry optimization was performed at the MS-CASPT2 level. The minimum energy structure obtained (Figure 4c) presents a slightly twisted dihedral angle of 172° and an elongated N–N distance that corresponds to a single bond. This structure is similar to the ππ* local minimum of azobenzene placed on the relaxation path along the torsional coordinate, but in the case of 3-PAPy this is the global ππ* minimum. In energetic terms, it is located 53.9 kcal·mol^−1^ above the ground-state minimum (see Table 3), on the S_1_ PES. A local minimum for this state was also found on S_1_ at a planar geometry (similar to the pedal-like global ππ* minimum of azobenzene) at 58.8 kcal·mol^−1^, indicating a probable competition between the relaxation paths to both geometries. 

A crucial difference is observed for 3-PAPy relative to azobenzene: in the case of 3-PAPy, the ππ* state is more stable than the nπ* one in the ππ* minima location (rotated and planar) with a gap between them of 19.8 and 11.6 kcal·mol^−1^, respectively. On the other hand, at the corresponding minima of azobenzene, the nπ* state is more stable than ππ*, with energy differences of 3.6 and almost 25 kcal·mol^−1^, so our results predict different luminescence properties for 3-PAPy and azobenzene.

#### 3.3.4. ^1^(n^2^π*^2^) State

The SA-CASSCF-optimized minimum geometry, confirmed at the MS-CASPT2 level, shows a rotated geometry (Figure 4d), very similar to the one of the minimum of the nπ* state. The energy for this species is 62.3 kcal·mol^−1^ relative to the ground-state minimum, with a gap to the nπ* state of around 10 kcal·mol^−1^. The oscillator strength of transition to the ground state is small, so the probability of radiative transition is predicted to be very low. These results are very similar to the ones obtained for azobenzene in our previous work.

### 3.4. Reaction Mechanism

#### 3.4.1. Isomerization along the Ground State

**Figure 5:**
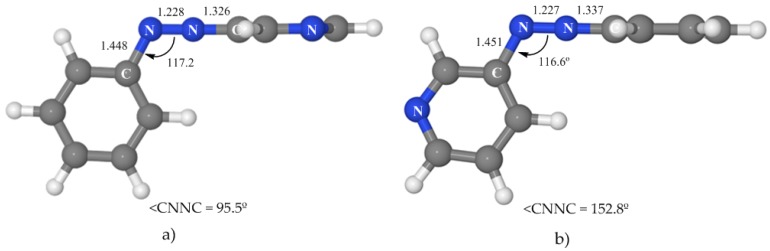
Optimized geometries for the transition states (TS) of the isomerization of 3-PAPy along the ground state following different reaction paths: (**a**) TS1 for inversion of the pyridine ring; (**b**) TS2 for inversion of the phenyl ring. Both geometries optimized at SA-CASSCF level.

The asymmetry of phenylazopyridine gives rise to two possible paths for the thermal isomerization along the ground-state surface that have been shown to follow a pure inversion mechanism. For both paths, the transition states correspond to structures where one N–N–C angle takes a value of 180°. The inverting ring also rotates onto itself due to the steric repulsion, adopting a perpendicular orientation relative to the molecular plane (see Figure 5). If this is the phenyl ring, the TS energy is 42.9 kcal·mol^−1^ above the global minimum while the barrier is slightly lower (39.0 kcal·mol^−1^) when the pyridine ring breaks the planarity of the molecule (Table 4). This difference means that this last path will be more probable for the thermal isomerization of 3-PAPy. No second-order saddle point connecting both TS was found.

#### 3.4.2. Isomerization along the nπ* State

**Figure 6:**
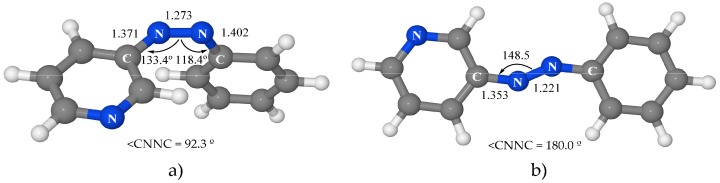
Optimized geometries of nπ*/S_0_ conical intersection. (**a**) Rotated minimum optimized at the SA-CASSCF level; (**b**) planar minimum located at the MS-CASPT2 level.

We have located a rotated and a planar nπ*/S_0_ conical intersection (see Figure 6 and Table 5) with geometries very similar to those of azobenzene. The rotated crossing point is near the nπ* minimum and located 50.6 kcal·mol^−1^ above the ground-state minimum. This means that it is 15.0 kcal·mol^−1^ below the vertical excitation to this state, what makes it easily accessible. On the other hand, the energy of the planar CI is 71.4 kcal·mol^−1^. If the initial absorption populates the nπ* state (at 65.6 kcal·mol^−1^), this crossing will be accessible only if the system has some extra energy. The consequence is that our results predict that 3-PAPy can also undergo isomerization on the nπ* surface if rotation is blocked. This hypothesis could be tested experimentally.

#### 3.4.3. Isomerization along the ππ* State

At the FC geometry, the topography of the PES of the ππ* excited state indicates that there are two possible relaxation coordinates that lead to stable structures—the slightly rotated global minimum and the planar local minimum. To confirm the preferential relaxation path, an MEP was run from the FC geometry on this state. Unlike for azobenzene, this path leads to the rotated minimum instead of the planar one. These results mean that this minimum is favored thermodynamically and kinetically, so it will be preferentially populated in comparison with the planar one. Given that both minima are located on the S_1_ PES, crossings with n_py_π* and nπ* states must occur along the relaxation path. 

The analysis of the results of the MEP from the FC geometry on the ππ* surface also provides the geometry of the crossing point between this state and the nπ* one. The structure obtained (Figure S5a of Supplementary Materials) is very similar to the rotated minimum except for a shorter N–N distance (1.351 Å versus 1.413 Å) with an energy of 66.1 kcal·mol^−1^. The strong oscillator strength for the transition to the ground state from the near ππ* minimum indicates that emission from the S_1_ ππ* species is possible. This deactivation path will then be competitive with the ππ*→nπ* internal conversion. This is a significant qualitative difference of 3-PAPy relative to azobenzene, where the quantum yield of this internal conversion was predicted to be one (in agreement with experimental measurements), while the luminescence from the ππ* rotated species was precluded. This difference means that the population of the nπ* state will be much more probable in azobenzene than in 3-PAPy.

The LIIC path calculated on the ππ* surface between the FC geometry and the planar minimum provides another ππ*/nπ* crossing point, of planar geometry (Figure S5b of Supplementary Materials), which is placed 69.7 kcal·mol^−1^ above the global minimum in a very similar region to the analogous crossing in azobenzene. This crossing may become relevant in geometrically constrained systems, but the efficiency of decay at this point is difficult to predict and it would be necessary to perform dynamic studies to provide an accurate prediction.

For an analogy with azobenzene, the conical intersection of the ππ* state with the n^2^π*^2^ state was also looked for along the rotation coordinate. The crossing point was located, but due to its high energy—106.3 kcal·mol^−1^—its involvement in the isomerization mechanism does not seem probable.

#### 3.4.4. Global Description of the Proposed Mechanism

**Figure 7:**
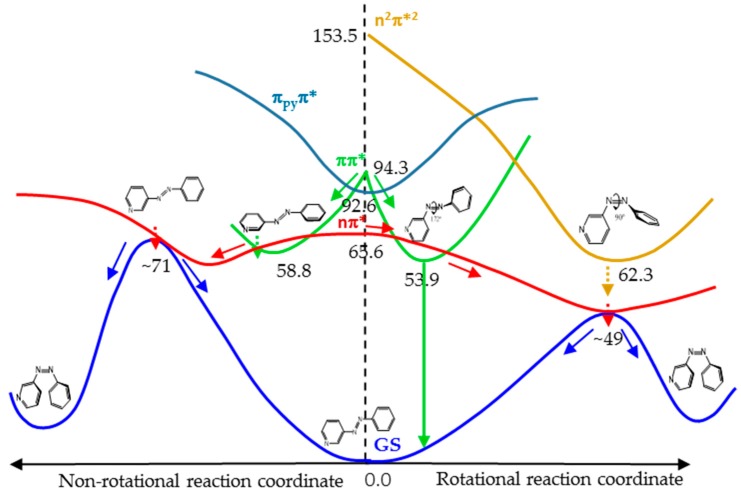
Global scheme of the profiles of the paths for the photoisomerization of 3-PAPy along the lower energy states for rotational and non-rotational paths (energies in kcal·mol^−1^, not to scale).

Figure 7 shows a cartoon scheme (not to scale) that summarizes the results obtained for 3-PAPy.

If the system is excited to the S_1_ (nπ*) state (weak absorption), the relaxation leads to the minimum energy structure on this surface, along the rotational coordinate, and to a conical intersection with the ground state in a neighboring geometry. After decay, the system can go back to the reactants or to the photoproduct, which is less stable but favored by the inertia of the movement of the nuclei. If this kind of system is excited to the ππ* state (strong absorption), it will relax towards the global rotated minimum, favored thermodynamically and kinetically, although a local pedal-like minimum can also be partially populated. In both relaxation paths, there are funnels for internal conversion to the nπ* state. The decay to this state is predicted to be slow in the surroundings of the pedal-like minimum, while it can be more efficient when following the rotational coordinate. As a whole, the quantum yield of the photoisomerization along the ππ* state is expected to be smaller than along the nπ* state.

For systems with constrained rotation, our results indicate that when the system is excited to the nπ* state, the quantum yield of photoisomerization along this PES will be smaller than for systems with free rotation, given that the planar nπ*/S_0_ conical intersection is located at higher energies in the former case. 

If the constrained system is excited to the ππ* state, it can only relax to planar geometries, in the direction of the pedal-like minimum. If this is reached, the system can decay radiatively from the pedal-like species. Along this relaxation path, it can also cross to the nπ* state. In this case, the kinetic energy accumulated from the initial absorption can be enough to reach the planar conical intersection with the ground state that will give rise to the *cis* photoproduct. Although the quantum yield of this process will not be very high, it is expected to be larger than when the system is initially excited to the nπ* state, given that the excess energy will be smaller in the latter case. 

For geometrical and energetic reasons, we do not expect the n_py_π* and n^2^π*^2^ states to be involved in the reaction mechanism in a significant proportion. This hypothesis was reinforced by the results obtained in the calculation of the profiles of the PES of the lowest energy states along the rotation and inversion reaction coordinates, which are shown in Figures S6 and S7 of Supplementary Materials. These figures show that the energy of the n_py_π* state is never, in fact, low enough to be involved in any mechanism of the photoisomerization reaction and that the probability to populate the n^2^π*^2^ state, when the system is excited to the nπ* or ππ* states, is quite low.

## 4. Conclusions

In this work we have studied the photoisomerization of 3-phenylazopiridine by ab initio calculations to elucidate the details of the reaction mechanisms that explain the experimental evidence compiled in the literature. The mechanisms proposed here are based on the topography of the potential energy surfaces determined computationally, using the SA-CASSCF/CASPT2 methodology.

Our computed vertical excitations for *trans*-3-PAPy show a good agreement with experimental results for transitions to the nπ* and ππ* states (64.3 kcal·mol^−1^ versus 65.6 kcal·mol^−1^ for nπ* state; 94.3 kcal·mol^−1^ versus 90.5 kcal·mol^−1^ for the ππ* state) and a poorer one for the n_py_π state (92.6 kcal·mol^−1^ versus 123.8 kcal·mol^−1^) due to the strong dependence of the energy of this state on the out-of-plane deformation of this system. On the other hand, the experimental spectrum has been reproduced satisfactorily. Generating a realistic sample and modelling the experimental environment are essential conditions for correctly simulating the real system. The geometrical deformations from the most stable structure have to be taken into account, given that they release the selection rules imposed by the symmetry and, therefore, give rise to a spectrum with more (weakly) allowed transitions and, consequently, with more absorption bands as, in fact, observed in the experimental spectrum. The strategy used by our group to do so has been validated with this calculation.

In comparison with azobenzene, phenylazopyridine presents an extra heteroatom that gives rise to a low energy ^1^(n_py_π*) excited state that does not exist in the former system and that could modify the photoreactivity of the latter one. In spite of this, our results show that, although the ^1^(n_py_π*) state is the second excited state at the Franck–Condon geometry, it will not be involved in the photoisomerization mechanism given that the minimum of this PES is located at a very high relative energy, making the n_py_π* species very unfavored thermodynamically.

Our results show that if 3-PAPy is excited to the nπ* state, in systems with free rotation, the mechanism of photoisomerization will be similar to that predicted for azobenzene along a rotational mechanism. If the system has the rotation constrained, our results indicate that the isomerization will take place through the planar nπ*/S_0_ CI that is localized very close to the analogous CI found in azobenzene. We predict that, in this case, the quantum yield of photoisomerization will be only slightly smaller for 3-PAPY than for azobenzene, given than the planar nπ*/S_0_ conical intersection is located at energies only slightly higher in 3-PAPy. No alternative and viable pathway has been found along an inversion path since the quasi-linear CI was localized well above the excitation energy to the ππ* state. 

If the initial absorption excites the system to the ππ* state, 3-PAPy can follow one of the same two competitive pathways as found for azobenzene, leading to the pedal-like minimum, or to the partially rotated global minimum. However, opposite to azobenzene, the preferential path in 3-PAPy leads to the rotated minimum. Another difference with azobenzene is that both minima are located on S_1_, so the quantum yield of the internal conversion from the ππ* state to the nπ* state will be smaller in 3-PAPy than in azobenzene. Relaxation paths to the pedal-like minimum and to the rotated one are considered to be highly competitive, especially in restricted cases. 

The path proposed for azobenzene through a conical intersection with n^2^π*^2^ is found to be much higher in energy in 3-PAPy, so it cannot be proposed as an alternative route.

Eventually, decay from the ππ* to the nπ* will take place through one of the accessible crossings, rotated or planar, depending on the inertia of the route and the restrictions applied. If the system is free to rotate, it will decay to the ground state through the rotated nπ*/S_0_ CI since it has a lower energy. If the rotation is constrained, the energy absorbed by the system previously excited to the ππ* state will be enough to reach the planar nπ*/S_0_ CI, while the isomerization after excitation to nπ* will take longer due to the larger energy barrier.

As a whole, the differences in photochemical properties between phenylazopyridine and azobenzene are not due to the presence of an extra N atom in the latter system (and extra low-lying excited states of n_py_π* character) but to small quantitative changes that modify the subtle equilibrium between competitive paths on the ππ* and nπ* potential energy surfaces. To get quantitative information about this competition, dynamic studies should be performed, but this is beyond the scope of this work.

## Figures and Tables

**Table 1:** MS-CASPT2 energies (in kcal·mol^−1^) relative to the ground-state minimum, for the three lowest excited states of 3-PAPy at the *trans* and *cis* ground-state minima. Energies in bold correspond to the optimized state. Experimental values in parentheses [18].

	*trans*-3-PAPy		*cis*-3-PAPy	
State	MS-CASPT2	Oscillator Strength	MS-CASPT2	Oscillator Strength
GS	**0.0**	**-**	**11.3**	-
^1^(nπ*)	65.6 (64.3)	<10^−5^	78.9	0.36 × 10^−1^
^1^(n_py_π*)	92.6 (123.8)	0.15 × 10^−3^	133.8	0.92 × 10^−2^
^1^(ππ*)	94.3 (90.5)	0.99	114.9	0.12
^1^(n^2^π*^2^)	153.5	<10^−4^	154.0	0.10 × 10^−1^

GS: ground state.

**Table 2:** Comparison of the computed and experimental [18] lowest absorption energies.

State	MS-CASPT2 (Single-Point)	MS-CASPT2 (Statistical)	Experimental
^1^(nπ*)	65.6	57.2	64.3
^1^(n_py_π*)	92.6	115.1	123.8
^1^(ππ*)	94.3	84.4	90.5
^1^(n^2^π*^2^)	153.5	146.4	-

**Table 3:** MS-CASPT2 energies (in kcal·mol^−1^) relative to the ground-state minimum, for the lowest states of 3-PAPy at the different excited-state minima. Energies in bold correspond to the optimized state.

	^1^(nπ*) State		^1^(n_py_π*) State	
**State**	**Relative Energy**	**Osc. Strength**	**Relative Energy**	**Osc. Strength**
GS	42.4		23.4	
^1^(nπ*)	**48.4**	**0.91 × 10^−3^**	83.9	<10^−7^
^1^(n_py_π*)	-	-	**75.7**	**<10^−5^**
^1^(ππ*)	110.7	0.43 × 10^−1^	95.1	0.87
^1^(n^2^π*^2^)	62.0	0.73 × 10^−3^	144.4	<10^−4^
	^1^(ππ*) state, rotated geometry		^1^(ππ*) state, planar geometry	
**State**	**Relative Energy**	**Osc. strength**	**Relative Energy**	**Osc. strength**
GS	6.6		7.7	
^1^(nπ*)	73.7	0.10 × 10^−1^	70.4	0.34 × 10^−2^
^1^(n_py_π*)	108.6	0.69 × 10^−2^	105.3	0.50 × 10^−2^
^1^(ππ*)	**53.9**	**0.45**	**58.8**	**0.48**
^1^(n^2^π*^2^)	146.2	0.22 × 10^−2^	139.1	0.49 × 10^−2^
	^1^(n^2^π*^2^) state			
**State**	**Relative Energy**	**Osc. strength**		
GS	46.0			
^1^(nπ*)	52.8	0.14 × 10^−3^		
^1^(n_py_π*)	-	-		
^1^(ππ*)	113.3	0.44 × 10^−1^		
^1^(n^2^π*^2^)	**62.3**	**0.75 × 10^−3^**		

**Table 4:** MS-CASPT2 energies relative to the ground-state minimum, for the four lowest excited states of 3-PAPy at the transition states geometries. Labelling according to Figure 4. Energies in bold correspond to the optimized state.

State	TS1	Osc. Strength	TS2	Osc. Strength
GS	**39.0**	-	**42.9**	-
^1^(nπ*)	67.7	0.30 × 10^−3^	66.3	0.23 × 10^−2^
^1^(n_py_π*)	145.6	0.51	152.4	0.71 × 10^−2^
^1^(ππ*)	133.9	0.25	137.1	0.65 × 10^−2^
^1^(n^2^π*^2^)	120.1	0.23 × 10^−1^	123.2	0.75 × 10^−2^

**Table 5:** MS-CASPT2 energies relative to the ground-state minimum, for the four lowest excited states of 3-PAPy at the rotated and planar nπ*/S_0_ conical intersections (CIs), both optimized at the SA-CASSCF level. Energies in bold correspond to the optimized state.

State	nπ*/S_0_ Rotated CI	Osc. Strength	nπ*/S_0_ Planar CI	Osc. Strength
GS	**48.0**	**-**	**71.1**	
^1^(nπ*)	**50.6**	0.11 × 10^−2^	**71.4**	0.27 × 10^−5^
^1^(n_py_π*)	133.1	0.11 × 10^−1^	152.8	0.20 × 10^−3^
^1^(ππ*)	120.0	0.29 × 10^−2^	141.0	0.30 × 10^−2^
^1^(n^2^π*^2^)	76.7	0.21 × 10^−2^	105.0	0.55 × 10^−4^

## Supplementary Materials

The following are available online at www.mdpi.com/1996-1944/10/12/1342/s1: Parameters of the force field that determine the topology of the methanol molecule in the MD calculations run with the Gromacs 4.5 package. Figure S1: Active orbitals. Figure S2. Test calculations using different basis sets for the relative energies of the first two singlet roots along the rotational path. Basis 1: 6-31G*; Basis 2: ANO-rcc-vdzp; Basis 3: ANO-rcc-vtzp. Figure S3: Distribution, in the geometries selected to reproduce the absorption spectra of 3-PAPy, of the values of the CNNC dihedral angle, the CNN angle and the N–N distance. Figure S4: ^1^(nπ*) optimized geometry at the SA-CASSCF level. Figure S5: nπ*/ππ* conical intersection geometries located at the MS-CASPT2 level at (a) rotated and (b) planar geometries. Figure S6. Energy profiles of the PES of the lowest states of PAPy along the main coordinate of the rotation mechanism path. Geometries optimized at the B3LYP level for the ground state at fixed values of the CNNC dihedral angle. Geometries calculated at the CASPT2(12,9) level. Figure S7. Energy profiles of the PES of the lowest states of PAPy along the main coordinate of the inversion mechanism path. Geometries optimized at the B3LYP level for the ground state at fixed values of the NNC angle. Geometries calculated at the CASPT2(12,9) level. Table S1: MS-CASPT2 energies (in kcal·mol^−1^) relative to the ground-state minimum of the lowest states of 3-PAPy at the nπ* state minima optimized at the SA-CASSCF level and at the MS-CASPT2 level.
