# Supplementary material for: Phenylazopyridine as Switch in Photochemical Reactions. A Detailed Computational Description of the Mechanism of Its Photoisomerization
Source: Materials (Basel). 2017 Nov 23;10(12):1342. doi: 10.3390/ma10121342 (PMC5744277; doi:10.3390/ma10121342)

# Supporting information

# Phenylazopyridine as switch in photochemical reactions. A detailed computational description of the mechanism of its photoisomerization

**Gerard Alcover [1], Josep Casellas [1], Coen de Graaf [1, 2] and Mar Reguero [1,*]**

[1] Departament de Química Física i Inorgànica, Universitat Rovira i Virgili, Carrer Marcellí Domingo 1, 43007, Tarragona, Spain

[2] Institució Catalana de Recerca i Estudis Avançats (ICREA), Passeig Lluís Companys 23, 08010 Barcelona, Spain

**Parameters of the force field that determine the topology of the methanol molecule in the MD calculations run with the Gromacs 4.5 package.**

| id | at type | res nr | res name | at name | cg nr | charge | mass |
|---|---|---|---|---|---|---|---|
| 1 | CT | 1 | SOL | C1 | 1 | 0.1166 | 12.01000 |
| 2 | OH | 1 | SOL | O2 | 1 | -0.6497 | 16.00000 |
| 3 | HO | 1 | SOL | H3 | 1 | 0.4215 | 1.00800 |
| 4 | HC | 1 | SOL | H4 | 1 | 0.0372 | 1.00800 |
| 5 | HC | 1 | SOL | H5 | 1 | 0.0372 | 1.00800 |
| 6 | HC | 1 | SOL | H6 | 1 | 0.0372 | 1.00800 |

FLEXIBLE

bonds

| i | j | funct | length | force_constant | length | force_constant |
|---|---|---|---|---|---|---|
| 1 | 2 | 1 | 0.14100 | 133977.6 | 0.14100 | 133977.6 |
| 2 | 3 | 1 | 0.10100 | 181707.1 | 0.10100 | 181707.1 |
| 1 | 4 | 1 | 0.10980 | 142351.2 | 0.10980 | 142351.2 |
| 1 | 5 | 1 | 0.10980 | 142351.2 | 0.10980 | 142351.2 |
| 1 | 6 | 1 | 0.10980 | 142351.2 | 0.10980 | 142351.2 |

angles

| i | j | k | funct | angle | force_constant | angle | force_constant |
|---|---|---|---|---|---|---|---|
| 1 | 2 | 3 | 1 | 108.0 | 230.274 | 108.0 | 230.274 |
| 4 | 1 | 2 | 1 | 109.5 | 146.538 | 109.5 | 146.538 |
| 5 | 1 | 2 | 1 | 109.5 | 146.538 | 109.5 | 146.538 |
| 6 | 1 | 2 | 1 | 109.5 | 146.538 | 109.5 | 146.538 |
| 4 | 1 | 5 | 1 | 109.5 | 146.538 | 109.5 | 146.538 |
| 4 | 1 | 6 | 1 | 109.5 | 146.538 | 109.5 | 146.538 |
| 5 | 1 | 6 | 1 | 109.5 | 146.538 | 109.5 | 146.538 |

dihedrals

| i | j | k | l | funct | angle | force_constant | m |
|---|---|---|---|---|---|---|---|
| 4 | 1 | 2 | 3 | 1 | 10.5 | 43.9614 | 2 |
| 5 | 1 | 2 | 3 | 1 | 10.5 | 43.9614 | 2 |
| 6 | 1 | 2 | 3 | 1 | 10.5 | 43.9614 | 2 |

**Figure S1.** Active orbitals.

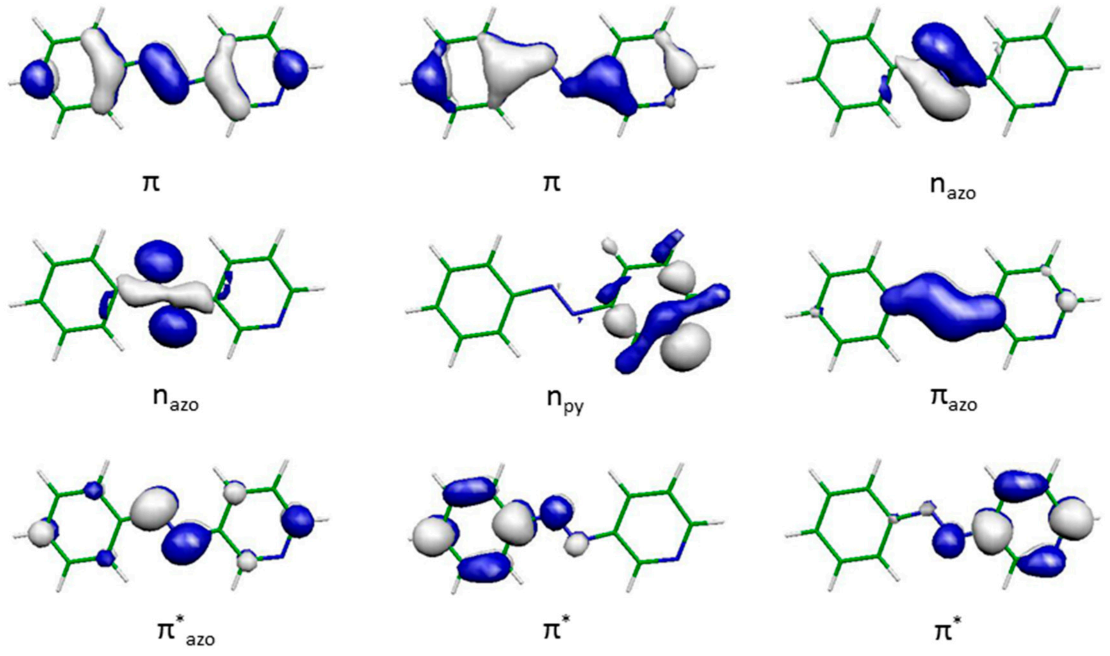


**Figure S2.** Test calculations using different basis sets for the relative energies of the first two singlet roots along the rotational path. Basis 1: 6-31G*; Basis 2: ANO-rcc-vdzp; Basis 3: ANO-rcc-vtzp.

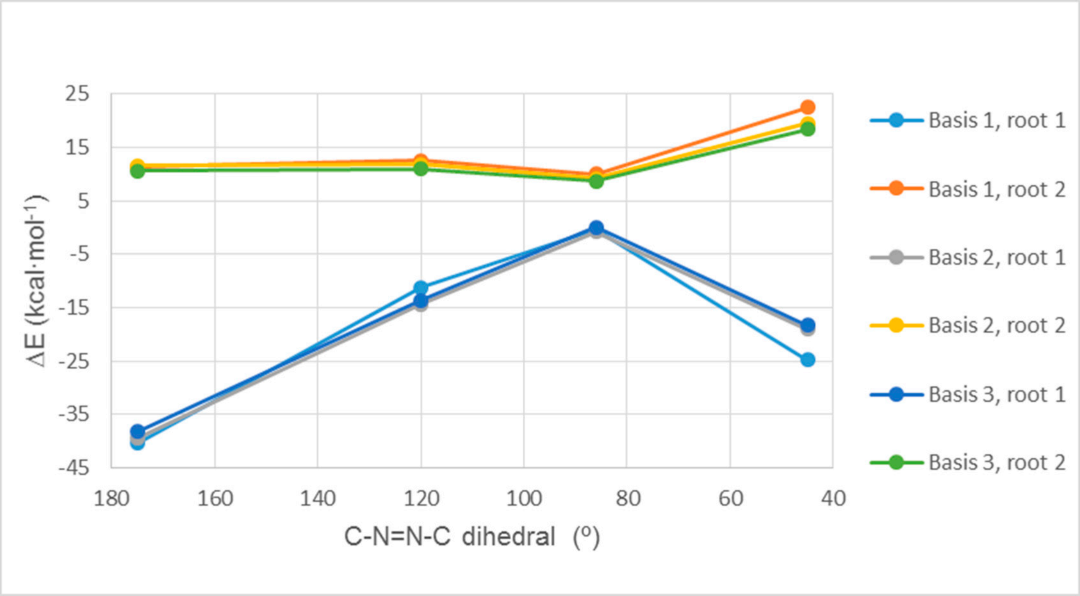

**Figure S3.** Distribution, in the geometries selected to reproduce the absorption spectra of 3-PAPy, of the values of the CNNC dihedral angle, the CNN angle and the N-N distance.

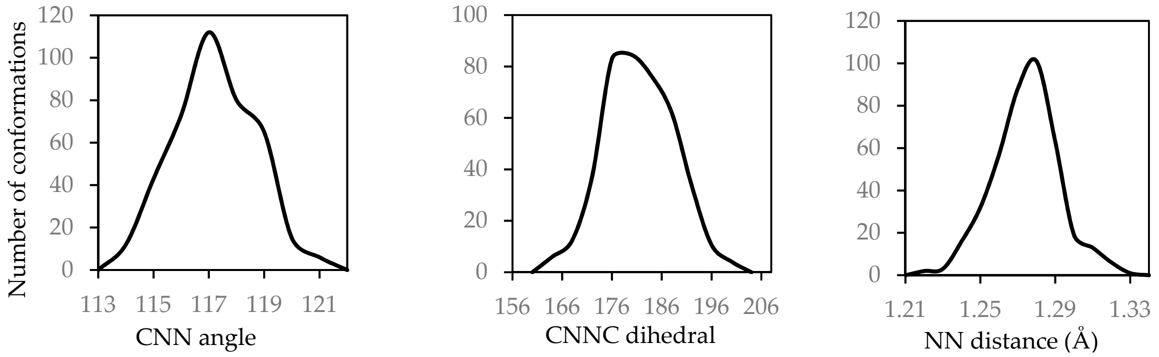


**Figure S4.** $^{1}$(n◎*) optimized geometry at CASSCF level.

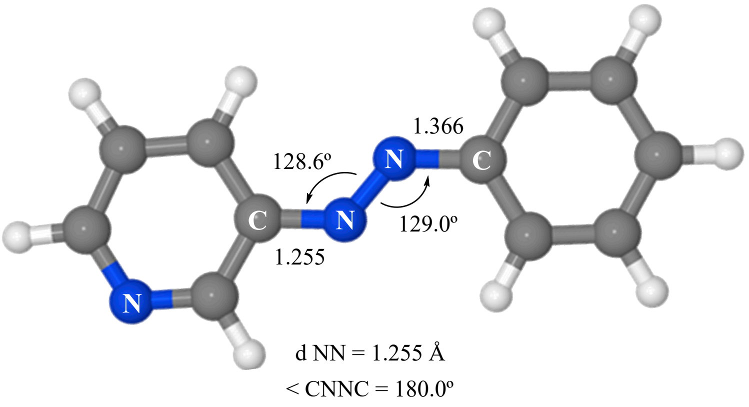


**Table S1.** MS-CASPT2 energies (in kcal·mol$^{-1}$) relative to the ground state minimum of the lowest states of 3-PAPy at the n◎* state minima optimized at CASSCF level and at MS-CASPT2 level.

| State | CASSCF optimization | Osc. strength | CASPT2 optimization | Osc. Strength |
|---|---|---|---|---|
| GS | 16.3 | | 42.4 | |
| **$^{1}$(n⚜*)** | **52.5** | **< $10^{-7}$** | **48.4** | **$0.91\cdot10^{-3}$** |
| $^{1}$(n$_{py}$◎*) | 100.5 | $0.31\cdot10^{-3}$ | - | - |
| $^{1}$(◎◎*) | 106.1 | 1.11 | 110.7 | $0.43\cdot10^{-1}$ |
| $^{1}$(n$^{2}$◎*$^{2}$) | 117.6 | $0.28\cdot10^{-3}$ | 62.0 | $0.73\cdot10^{-3}$ |

**Figure S5.** n◎*/◎◎* conical intersection geometries located at MS-CASPT2 level at (a) rotated and (b) planar geometries.

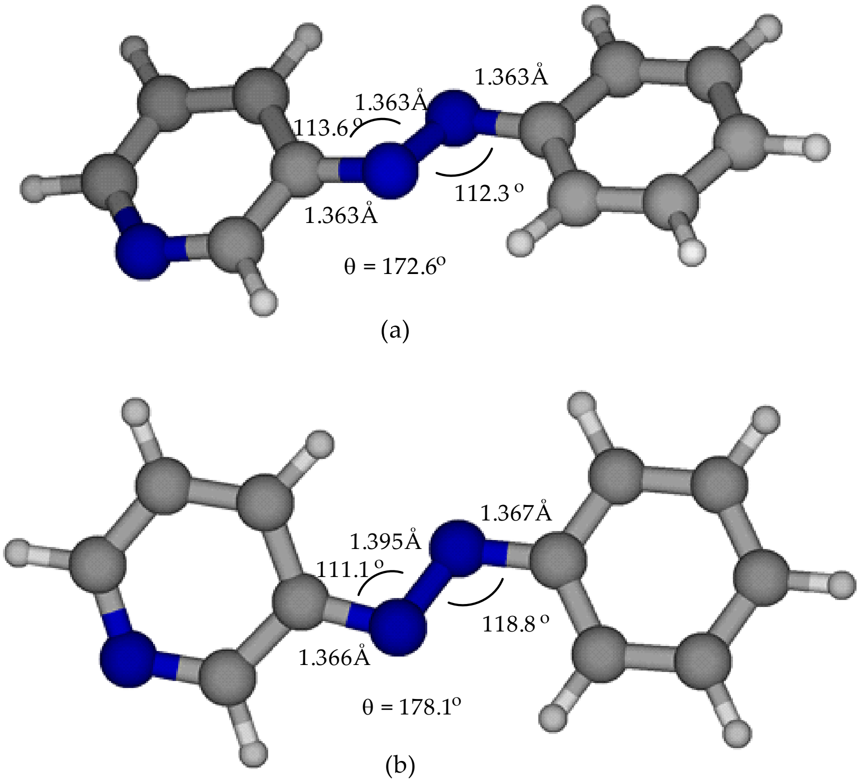


**Figure S6.** Energy profiles of the PES of the lowest states of PAPy along the main coordinate of the rotation mechanism path. Geometries optimized at B3LYP level for the ground state at fixed values of the CNNC dihedral angle. Geometries calculated at CASPT2(12,9) level.

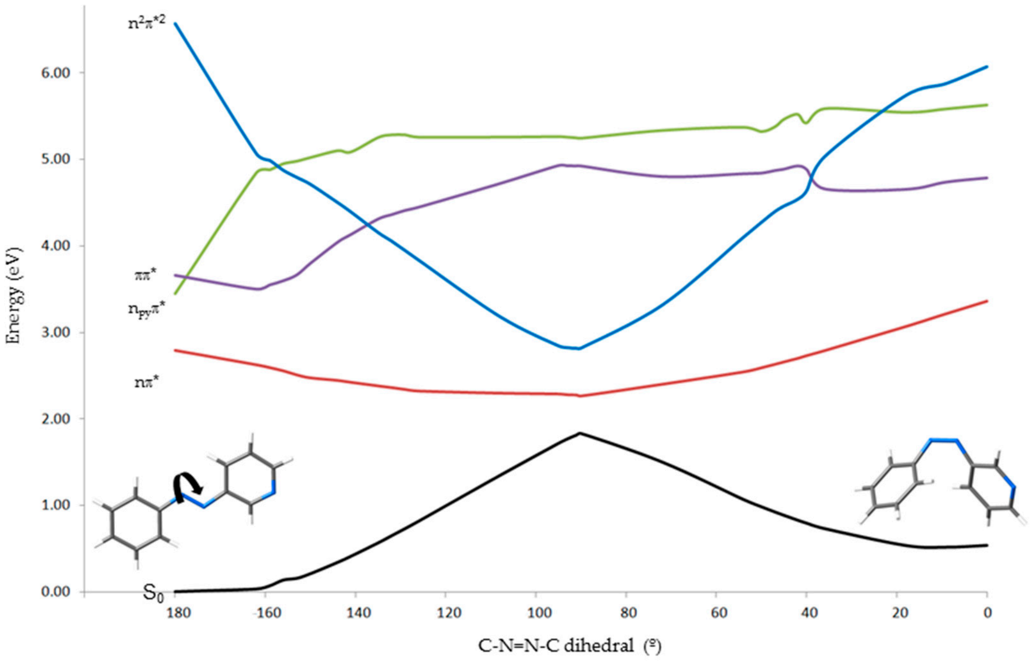

**Figure S7.** Energy profiles of the PES of the lowest states of PAPy along the main coordinate of the inversion mechanism path. Geometries optimized at B3LYP level for the ground state at fixed values of the NNC angle. Geometries calculated at CASPT2(12,9) level.

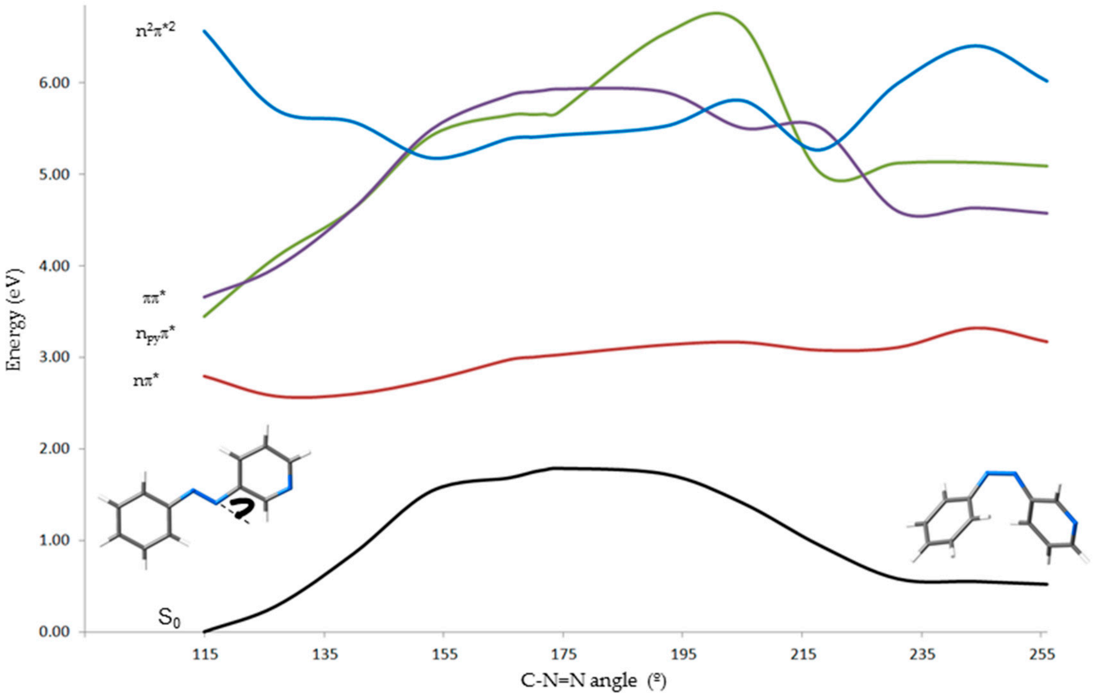

Supplement: Supplementary file 1 [file materials-10-01342-s001.pdf]
